# Microstructure and Properties of ZrB_2_-SiC Reinforced Copper Matrix Composite Coatings Prepared by Laser Cladding

**DOI:** 10.3390/ma15196777

**Published:** 2022-09-30

**Authors:** Yuehong Zhao, Zaiji Zhan, Xiangzhe Lv, Haiyao Cao

**Affiliations:** 1 Key Laboratory of Metastable Materials Science & Technology, Yanshan University, Qinhuangdao 066004, China; 2 Mechanical and Electrical Engineering Department, Qinhuangdao Vocational and Technical College, Qinhuangdao 066100, China

**Keywords:** laser cladding, in-situ synthesis, ZrB_2_-SiC, copper, wear

## Abstract

With the use of electrolytic Cu powder, Zr powder, Si powder and nickel-coated B_4_C powder as cladding powders, in-situ synthesized ZrB_2_-SiC reinforced copper matrix composite coatings were prepared by laser cladding on the surface of the copper substrate to improve the surface hardness and wear resistance. Under the condition of a laser energy density at 60 kJ/cm^2^, the macroscopic surface of the composite coating was continuously flat. The microstructure and phase of the cladding coating were analyzed by means of XRD and SEM. The reinforcements with nano-scale particle and micron-scale needle-like structures were in-situ synthesized in the cladding coating, and the content of the reinforcement phase decreased slightly from the coating surface to the substrate. The phase analysis results showed that the reinforcements included ZrB_2_ and SiC. When the content of the reinforcement was increased to 30 wt%, microhardness also increased from 48 HV_0.2_ to 309 HV_0.2_, which was about 5.6 times that of the copper matrix. The wear resistance of the composite coatings was characterized by current-carrying wear tests. By keeping the sliding speed and load constant, the wear rate decreased with an increase in the reinforcement content, and the wear mechanism changed from adhesive wear to abrasive wear. The wear rate of the composite coating with the current was higher than that without the current due to its electric ablation and high temperature.

## 1. Introduction

Copper is widely used as brushes, bearing bushings and contact wires in thermal and electronic applications [1,2] because of its high electrical and thermal conductivity, good corrosion resistance and ease of fabrication [3,4]. However, lower mechanical properties and poor wear resistance limit the extensive application of pure Cu. Some research work has been carried out to improve the mechanical and tribological properties of copper so as to prolong its service life and reduce operating costs [5]. Laser cladding is one of the metal deposition processes in which a powder coating is deposited onto the substrate material, and the two materials are fused by metallurgical bonding through the action of the laser beam [6,7]. The method has been used to manufacture a coating on the surface of metal materials such as titanium alloy and steel [8,9,10,11,12,13]. Laser cladding technology has become a research hot topic in recent years. In-situ synthesis technology is also a new technology for preparing composite materials, which has been developed in recent years. Its principle is to generate one or several reinforced phase particles from different elements or reactants via chemical reactions under certain conditions in the metal matrix so as to improve the properties of the composites [14]. The combination of in-situ synthesis technology and laser cladding offer advanced benefits and have been applied widely. For example, Cu-TiB_2_ composite coatings on the pure copper surface have been synthesized by in-situ synthesis and laser cladding [15]. An in-situ synthesis of the ceramic particle reinforced phase was generated by laser cladding Ni-based composite coatings on the copper alloy surface [16]. Therefore, laser cladding is an advanced technology suitable for preparing wear-resistant coatings on copper surfaces.

In this paper, the methods of in-situ synthesis and laser cladding were adopted to improve the surface hardness and wear resistance of the copper substrate. By fully mixing the electrolytic copper powder with Zr powder and Si powder with Ni package B_4_C powder, the chemical reactions occurred by means of laser cladding. ZrB_2_ was an ultra-high temperature ceramic which showed exceptional multifunctional properties, including an increased hardness, melting point, electrical conductivity, and wear resistance. The ZrB_2_-SiC reinforced phase was generated in situ, and no cracks were seen at the interface due to the excellent wettability between the reinforced phase and matrix. Moreover, the ceramic-reinforced phase offered not only superior hardness but also excellent wear resistance, which was beneficial for improving the service characteristics of the material [17,18]. In the previous work, ZrB_2_-ZrC reinforced copper-matrix composite coatings were successfully prepared by laser cladding [19], and the wear resistance of the coating was tested under low-speed reciprocating friction conditions. In this work, the high-speed, current-carrying wear resistance of ZrB_2_-SiC reinforced copper-matrix composite coatings were tested and analyzed in detail.

## 2. Experimental Materials and Methods

The substrate was pure copper (the grade of T2), its size was a cuboid of 150 × 50 × 15 mm^3^, and the surface was required to be flat without deformation. The surface of 150 × 50 mm^2^ was selected as the experimental surface.

The experimental surface was polished with 150# sandpaper to remove the oxide layer and impurities from the surface. Then the surface was cleaned using acetone and anhydrous ethanol to remove oil stains and was blow-dried after cleaning.

The selected cladding powders include Cu powder, Si powder and Ni package B4C (B_4_C contents 60wt%) powder. The specific parameters of the cladding powders (morphology, purity, and particle size) are listed in Table 1.

The method of in-situ synthesis was used. The ratio of cladding powders was calculated according to the in-situ reaction equation:

2Zr + Si + B_4_C = 2ZrB_2_ + SiC (1)

from the formula above, and the powder ratio of Zr:Si:B_4_C was obtained as 2:1:1 in the molar. After mixing the powders accordingly, the mixture was used as the reinforcing phase and then blended with electrolytic pure copper powder.

The pre-mixed powders were treated by using an XQM-21 variable frequency planetary ball mill at the rotation speed of 140 r/min for 60 min. In order to avoid oxidation, argon gas was used for protection over the ball mill, and the ball-to-power ratio was 3:1. The mixed powders were then placed in an oven to dry at 100 °C for 100 min. The experiment was performed on a Fiber coupling output all-solid-state laser with a wavelength of 1.06 μm. The synchronous lateral powder-feeding method was adopted to feed the powders at the speed of 2 g/min, and the carrier gas and protective gas were argon.

The substrate plate was fixed onto the three-dimensional moving platform. Due to the large amount of heat that could transfer to the copper substrate plate, the substrate was preheated by laser irradiation to narrow the temperature difference between the reaction pool and substrate. A laser power of 1200 W was used to illuminate the surface of the substrate for 4 min, which brought the substrate temperature to about 400 °C before the laser processing. The laser power was set between 1800 and 2300 W, the light spot diameter was 3 mm, and the scanning velocity was 1 mm/s. The laser energy density was in the range of 40.00–73.33 kJ/cm^2^.

The sample was cut out by a wire electric discharge machine (EDM, Beijing Ninghua Technology Co., Ltd., Beijing, China, NH7732A), and the surface was polished with sandpaper, cleaned, and reserved. The cross-section of the laser cladding composite coating was analyzed by a D/Max-2500PC X-ray diffractometer using Cu target Kα radiation with a scanning step length of 0.02°, a scanning speed of 4°/min, and angle range of 10~100°. The etched reagent was composed of FeCl_3_ (5 g), HCl (37%, 10 mL), and H_2_O (80 mL). The surface of composite materials and the distribution of interface bonding, microstructure, tensile fracture morphology and the reinforcing phase of the composite coating were observed and analyzed using the KYKY-3200 scanning electron microscope (SEM). A NORAN SYSTEM7 energy spectrum analyzer was used in conjunction with SEM, which could conduct a quantitative and qualitative analysis of elements. The characteristics of the samples were observed by transmission electron microscopy (TEM, FEI’s Tecnai G2 F30 S-TWIN).

**Figure 1:**
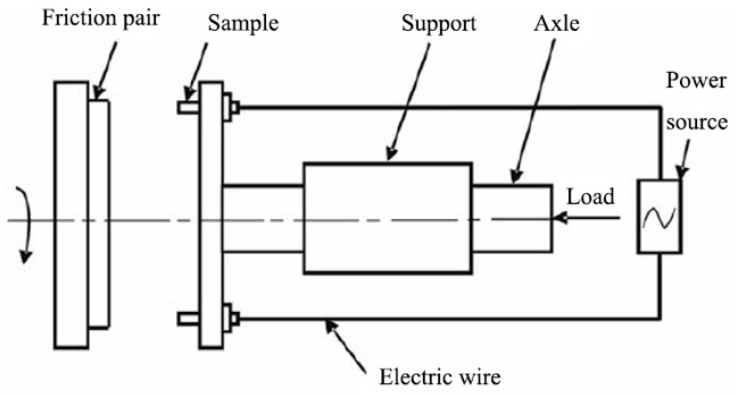
Schematic diagram of high-speed, current-carrying test platform.

The surface microhardness of the coating was measured using HVS-1000 Vickers microhardness with a load of 2 N and the holding time of 10 s, and the average microhardness was calculated from 5 test points. The tribological property of the samples was investigated using a high-speed loading MMS-1.5GZ wear tester, as shown in Figure 1. The pin was made of the test materials, and the counter plate was made of 52,100 steel (HRC61). The sliding speed was in the range of 70–110 m/s with a sliding time of 300 s, a load of 50 N, and the loaded current was 0 or 20 A. In the previous work, ZrB_2_-ZrC, which reinforced copper matrix composite coatings, was successfully prepared by laser cladding [19], and the wear resistance of the coating was tested under low-speed reciprocating friction conditions. In this work, the high-speed, current-carrying wear resistance of ZrB_2_-SiC reinforced the copper matrix composite coating and was tested and analyzed in detail.

## 3. Thermodynamic Calculation

The elements in the laser cladding reaction system included Zr, Si, B_4_C and Cu powders. The mixed powders were melted on the surface of the matrix by a laser to form a molten pool, and an in-situ synthesis reaction occurred in the molten pool to generate the enhanced phase. In order to ensure the expected reinforcement phase was synthesized, it was necessary to analyze the possible chemical reactions. Those reaction equations were as follows:

B_4_C = 4B + C (2)

Zr + 2B = ZrB_2_ (3)

Si + C = SiC(4)

Si + 6B = SiB_6_ (5)

Zr + C = ZrC(6)

Zr + Si = ZrSi(7)

The feasibility of the reaction can be predicted by calculating the Gibbs free energy (ΔG) of the chemical reaction. When the free energy value is negative, the reaction can proceed spontaneously [19].

**Figure 2:**
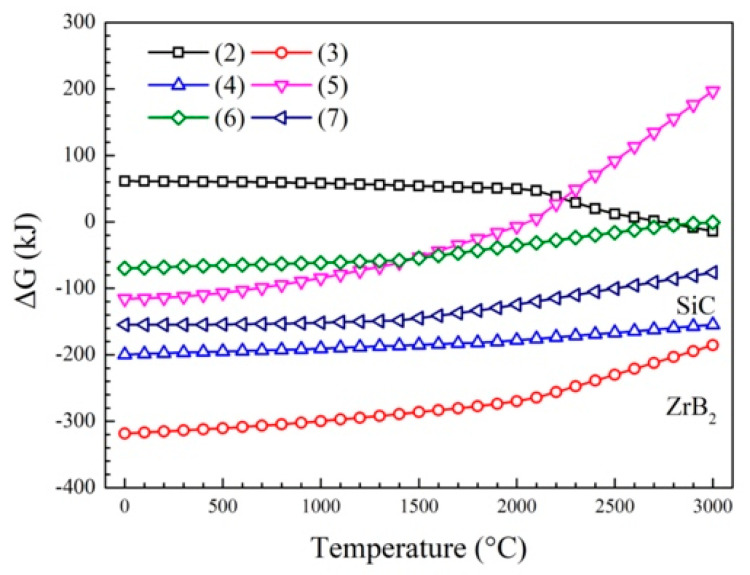
ΔG-T curve of the reactions.

According to the material thermochemical data manual [20,21] and thermodynamic calculation software HSC9.0, the Gibbs free energy of the possible reaction against the temperature was expressed by software, ΔG-T curves, as shown in Figure 2. In the range of 0–3000 °C, the free energies of reactions (3) and (4) were both negatively and significantly lower than those of other reactions, indicating that their synthetic phases ZrB_2_ and SiC would be preferentially synthesized under close-to-equilibrium conditions.

## 4. Results and Discussion

### 4.1. The Influence of Laser Energy Density on Surface Morphology of Cladding Coating

The surface morphology of the laser cladding composite coating could be regarded as the most direct manifestation of its performance. The purpose of the experiment was to obtain a continuous, flat, and uniform thickness of the cladding coating.

**Figure 3:**
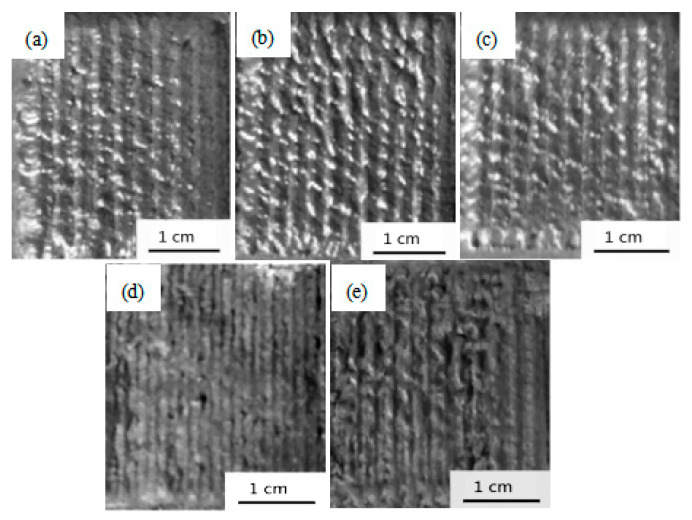
The surface morphology of cladding coating under different laser energy densities. (**a**) 46.67 kJ/cm^2^; (**b**) 53.33 kJ/cm^2^; (**c**) 60.00 kJ/cm^2^; (**d**) 66.67 kJ/cm^2^; (**e**) 73.33 kJ/cm^2^.

Figure 3 shows the surface morphology of the cladding coating of different energy densities with a reinforcement content of 30 wt%. Table 2 indicates the effect of the laser energy density on the roughness, thickness, and interface of the coating. With the increase in energy density, the coating surface morphology became flatter and more uniform (Figure 3a–c). An optimal state was obtained under the energy density of 60.00 kJ/cm^2^, the roughness (Rv) was decreased from 579 to 355, and the corresponding thickness of the coating was decreased from 2.1 mm to 1.8 mm. Further increasing the energy density to 66.67 kJ/cm^2^ or above, a thin coating with surface defects was observed (Figure 3d,e). Under a high laser energy density of 73.33 kJ/cm^2^, the roughness of the coating surface was increased because of the vaporization and splashing of the powders and the extensive melting of the substrate.

**Figure 4:**
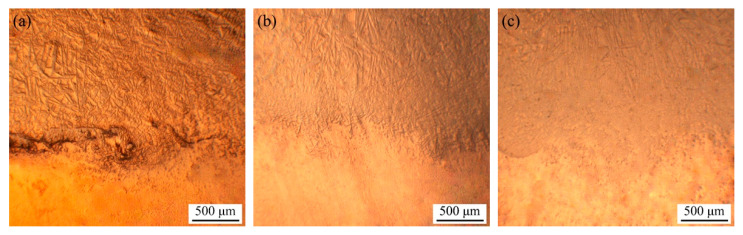
Interface bonding of composite coating at different energy densities. (**a**) 53.33 kJ/cm^2^; (**b**) 60.00 kJ/cm^2^; (**c**) 66.67 kJ/cm^2^.

Figure 4 shows the interfacial bonding conditions of the composite coatings under different energy densities. At a lower power density of 53.33 kJ/cm^2^ (as shown in Figure 4a), there were large defects at the interface, which indicated that the interface bonding was weak and insecure, and the coating was shedding at a lower power density of 40.00 kJ/cm^2^. When the energy density reached 60.00 kJ/cm^2^ (as shown in Figure 4b), the bonding between the composite coating and the substrate was better, there was no obvious defect, and the needle crystal in the coating basically grew along the direction of the vertical interface. As the energy density increased to 66.67 kJ/cm^2^ (as shown in Figure 4c), the interface bonding was uneven, the dilution effect of the coating on the substrate was greatly enhanced, and there were some defects at the interface.

### 4.2. The Phase Identification of Laser Cladding Composite Coating

**Figure 5:**
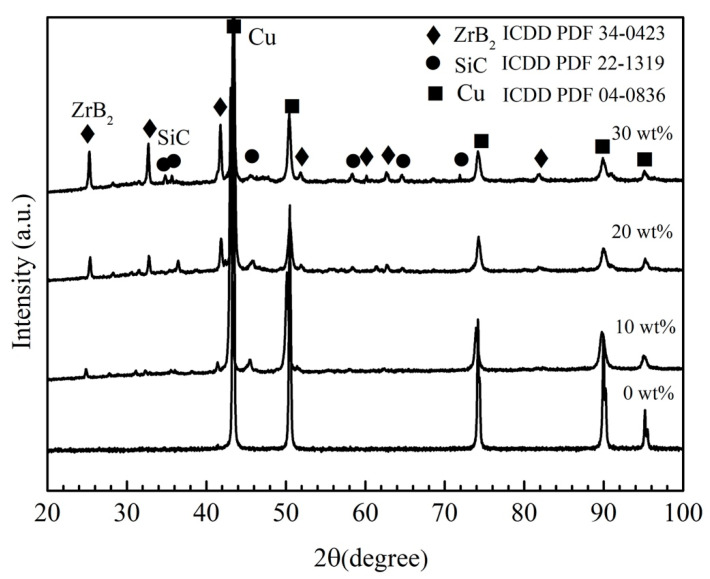
The X-ray diffraction phase analysis of laser cladding composite coating.

The phase analysis of the composite coating was carried out by XRD, and the obtained results are shown in Figure 5. Cu (PDF 04-0836), ZrB_2_ (PDF 34-0423), and SiC (PDF 22-1319) were detected in the laser cladding composite coating. Cu was the matrix phase of the composites, and both ZrB_2_ and SiC were in-situ synthesized reinforcement phases. No raw materials (Zr, Si and B_4_C) or additional phases were found. The results showed that the in-situ reinforcements were successfully formed in the molten pool over laser cladding. The synthesis reaction was completed, and no impurity phase was formed. This was in agreement with previous thermodynamic calculations. In addition, the diffraction peaks of ZrB_2_ and SiC were enhanced with the increase of the reactants content (from 10 wt% to 30 wt%).

### 4.3. The Microstructure of Laser Cladding Composite Coating

**Figure 6:**
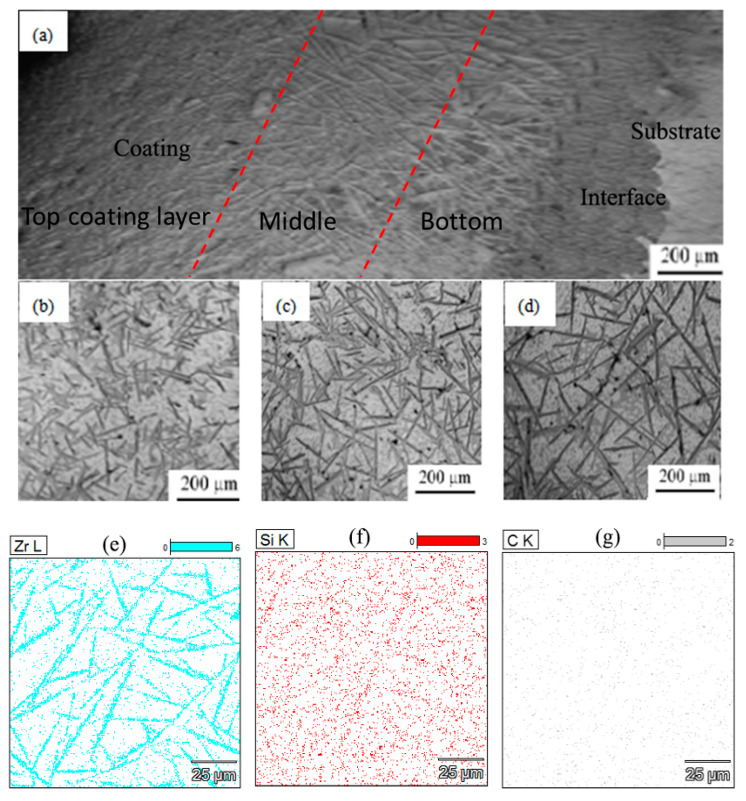
The micro-structure of laser cladding composite coating (**a**) Overall morphology; (**b**) Top; (**c**) Middle; (**d**) Bottom. The element distribution of (**e**) Zr, (**f**) Si, and (**g**) C.

The metallographic structure of the longitudinal section of the composite coating is shown in Figure 6. The composite coating was prepared under the energy density of 60.00 kJ/cm^2^, and the enhanced phase content was 20 wt%. From Figure 6a, there were a large number of needle-like phases in the coating zone, the overall structure of the cladding coating was dense, and no obvious defect was seen at the interface, so an excellent metallurgical bond was obtained between the coating and the substrate. The interface was not a flat, straight line, which indicated that the substrate had a certain dilution effect over the action of laser cladding.

According to the results of the XRD in Figure 5, there is a phase of Cu, ZrB_2_ and SiC in the coating, combined with the EDX analysis of element distribution (Figure 6e–g), meaning it could be confirmed that the needle-like phase was ZrB_2_. In addition, there were a few particles the same size as the diameter of the needle-like ZrB_2_, which might be the tip of the needle-like ZrB_2_.

A large number of needle-like micro-structures (ZrB_2_) could be observed within the composite coating, and there were obvious differences in size between the top, middle, and bottom of the coating. Figure 6b shows the microstructure at the top of the coating. It can be seen that the distribution of the fine needle-like ZrB_2_ was relatively uniform, and its length was about 100 μm. Figure 6c shows the structure of the middle cladding layer: the size of the ZrB_2_ phase was greater than 200 μm, which was larger than the one at the coating surface, and the distribution of needle-like phases was more uniform. Figure 6d shows the distribution of reinforcements in the bottom zone of the coating: the size of the ZrB_2_ phase was further increased and became needle-like phases with a width of 20 μm and length of about 400 μm.

During the laser cladding process, a molten pool was formed on the top of the substrate under laser irradiation, and the powders in the molten pool would react once the reaction condition was reached. Because the density of the reactant B_4_C was lower than that of the liquid copper, they easily floated to the top of the molten pool, and the enrichment phenomenon of the boron element and carbon element was formed at the surface. The Zr reacted with the B_4_C to form the ZrB_2_ phase. At the same time, the heat dissipation rate at the top of the coating was high, and the corresponding undercooling was also relatively high, so ZrB_2_ nucleated at a high rate in the surface coating. As a result, the number of ZrB_2_ phases at the surface was high, but the size was fine. Whereas the amount of ZrB_2_ phases at the bottom was limited, and the heat dissipation of the bottom was also slow, so the nucleation became difficult, but it was beneficial to the growth of the crystal nucleation, and therefore, the ZrB_2_ phase at the bottom was a large rod phase. The middle layer of the coating had sufficient time to cool, and its undercooling was not as large as the surface layer of the coating, so the size that the ZrB_2_ formed was longer, and the distribution was more uniform than that of the surface layer. In the laser cladding composite coating, this large needle-like ZrB_2_ was named primary ZrB_2_.

**Figure 7:**
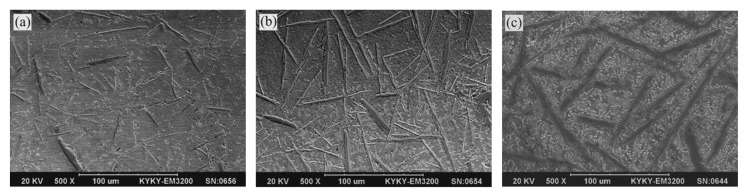
Microstructure on the top of the laser cladding coating (30 wt%) under different laser energy densities (**a**) 53.33 kJ/cm^2^; (**b**) 60.00 kJ/cm^2^; (**c**) 66.67 kJ/cm^2^.

Figure 7 shows the microstructural changes on the top of the coating at different laser densities. With the increase of the laser energy density from 53.33 kJ/cm^2^ to 66.67 kJ/cm^2^ took place, the length of the needle-like phase within the composite was observed to increase from about 40 μm to 120 μm. The average diameter of the needle-like phase was increased from about 3 μm to 10 μm. Increasing the laser energy density, the energy and the lifetime of the molten pool increased, and the cooling rate of the molten pool decreased, leading the size of the in-situ synthesized ZrB_2_ to increase.

**Figure 8:**
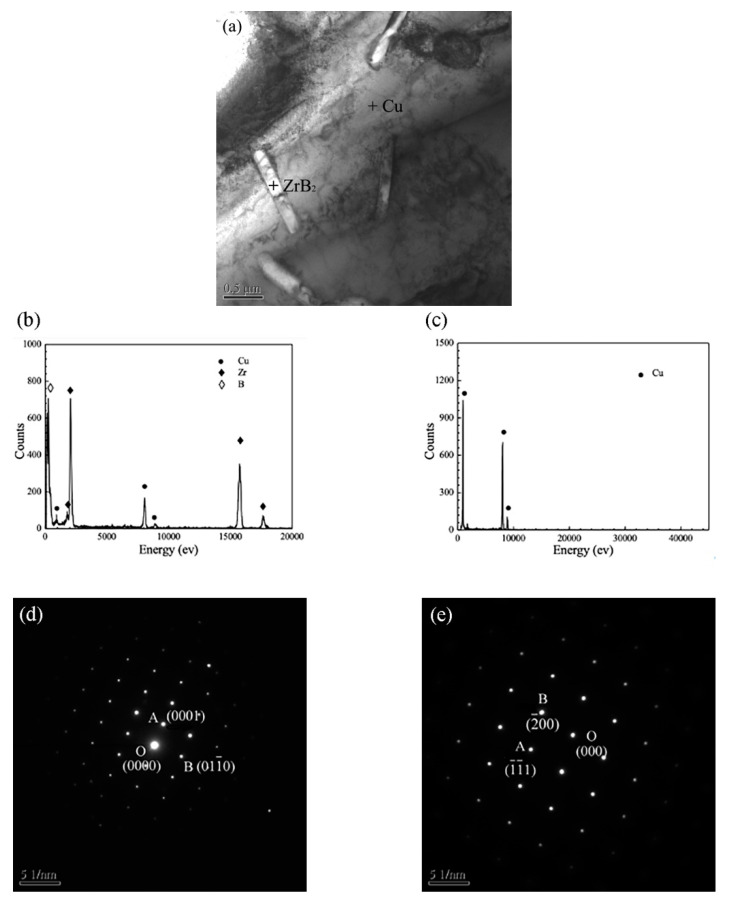
(**a**) TEM images of the composites (20 wt%, 53.33 kJ/cm^2^). EDX of (**b**) ZrB_2_ and (**c**) Cu. Selected area diffraction patterns of (**d**) small-sized secondary ZrB_2_ and (**e**) Cu.

The microstructure of the composites was further analyzed by TEM. As shown in Figure 8a, some small-sized, needle-like phases with diameters of about 200 nm and lengths of about 700 nm were observed in the composites prepared at a laser energy density of 53.33 kJ/cm^2^. Figure 8b shows the EDX result of the needle-like phase, which mainly contained Zr and B elements. The matrix contained the Cu element according to the EDS results of Figure 8c. Furthermore, selected area diffraction patterns (SADP) of the needle-like phase and Cu matrix were performed on the marked positions, and the results are shown in Figure 8d,e, respectively. From the measurements and calibrations of SADP, the crystal plane of ZrB_2_ was determined. Therefore, the small-sized, needle-like phase was secondary ZrB_2_.

**Figure 9:**
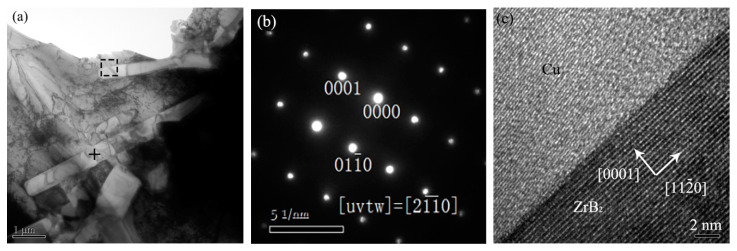
(**a**) TEM images of the composites (20 wt%, 66.67 kJ/cm^2^). (**b**) Selected area diffraction patterns of ZrB_2_. (**c**) High-resolution TEM of the interface of ZrB_2_ with Cu matrix.

As shown in Figure 9a, under the high laser energy density (of 66.67 kJ/cm^2^), small-sized ZrB_2_s with a diameter of about 500 nm and a length of more than 5 μm could be observed. The electron diffraction spots of the marked needle-like phase are shown in Figure 9b, where the needle-like phase was determined to be ZrB_2_ by measuring and labeling. The interface of reinforcements and the matrix was an important microstructure within the composites. Figure 9c shows the high-resolution TEM of the interface of ZrB_2_ and Cu. It was identified that ZrB_2_ and Cu were directly bound together without defects and other phases at the interface, indicating that the in-situ synthesized ZrB_2_ formed an excellent bond with the copper matrix. After measurement and calibration, the directions [0001] and [11$\bar{2}$0] of ZrB_2_ were determined as shown by the arrows.

**Figure 10:**
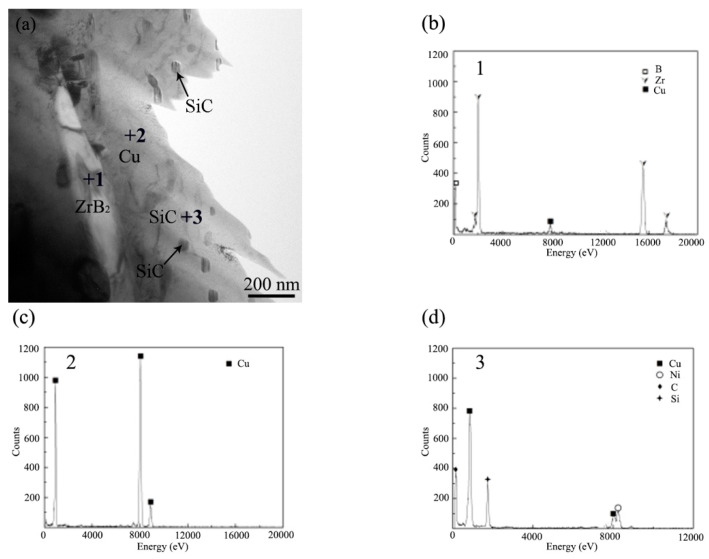

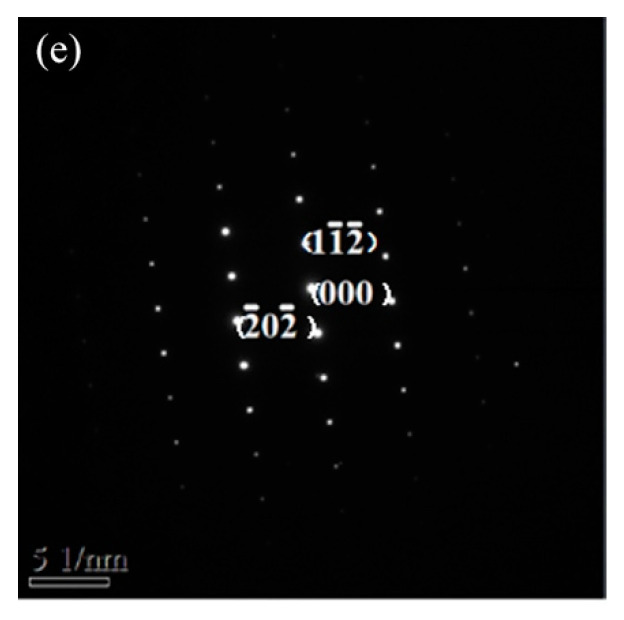
(**a**) TEM images of the composites (20 wt%, 66.67 kJ/cm^2^). EDS result of (**b**) ZrB_2_, (**c**) Cu and (**d**) SiC. (**e**) Selected area diffraction patterns of SiC.

Figure 10a shows the microstructure of the composites, the needle-like phase, and a large amount of the particles nanophase, which could be seen in the matrix. According to the EDS of spots 1 and 2 in Figure 10b,c, the needle-like phase was ZrB_2_, and the matrix was Cu. Figure 10d shows the EDS result of the particles nanophase (spot 3), which mainly contained Si, C and Cu elements. Due to the small size of the particle phase, Cu in the matrix was also detected by EDS at the same time. The SADP of the marked position is shown in Figure 10c, where the particle nanophase was SiC by measuring. The SiC was a nanoscale particle, so it was not clearly observable in the SEM image, while it could be detected in XRD and EDX due to the large number.

It was noted that three kinds of reinforcing phases were simultaneously synthesized in situ in the copper matrix: the nano-scale particle SiC (zero-dimensional), the nano-scale needle-like ZrB_2_ (one-dimensional) and the micron-scale primary ZrB_2_. On the one hand, because the reinforced phase, which possessed different sizes and shapes, showed different strengthening effects, and there was a synergistic strengthening effect between the reinforcements, the mechanical properties of the composite materials were significantly improved. On the other hand, two reinforcing phases with different morphology and different dimensions were uniformly distributed in the metal matrix, which could avoid the aggregation of the same kind of reinforcements and optimize the microstructure [22].

### 4.4. The Influence of Laser Energy Density on Hardness

As shown in Table 3, the microhardness of the composite coating (20 wt%) decreased from 184 HV_0.2_ to 167 HV_0.2_ with the increase of the laser energy density from 60 kJ/cm^2^ to 70 kJ/cm^2^. This was mainly due to the change in reinforcement size, which would have affected the mechanical properties of the composite coating. With the laser energy density increase from 60 kJ/cm^2^ to 70 kJ/cm^2^, the particle sizes of SiC increased from 25 nm to 40 nm; the length of ZrB_2_ increased from 35 μm to 80 μm. According to the dispersion strengthening mechanism [23], the smaller the particle size, the greater the resistance to the movement of dislocations, meaning that a higher strengthening efficiency is obtained with a smaller size reinforcement. Therefore, when the size of the reinforcement decreased, the stiffness of the composite increased.

**Figure 11:**
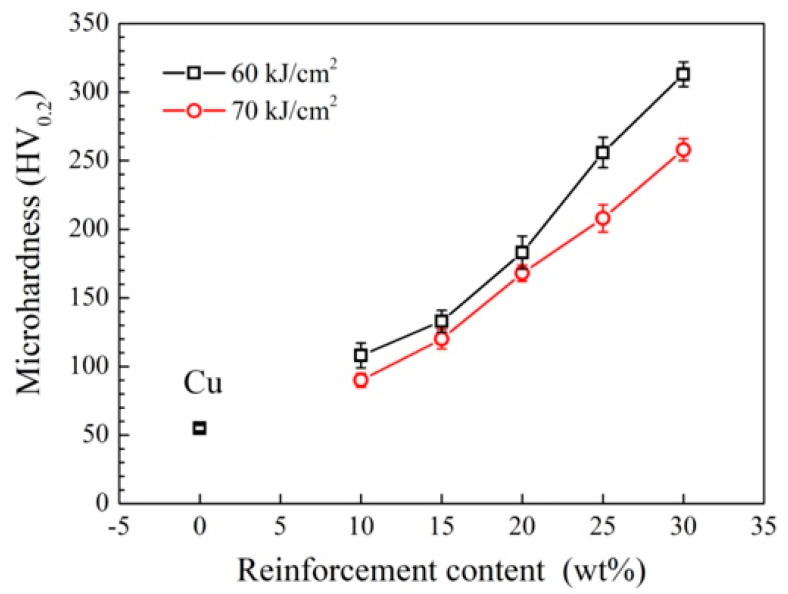
Change curve of the average microhardness of the composite coating with the reinforcements content.

Figure 11 shows the variation in the average microhardness of the composite coating with the content of the reinforcing phase. For the composite coatings prepared at 60 kJ/cm^2^, due to the reduction of the size of the acicular ZrB_2_ ceramic reinforcing phase, the strengthening efficiency was increased, and the microhardness of the composite coating increased. The higher the content of the reinforcing phase, the more obvious the increase in hardness. For example, when the reinforcement content was 10 wt%, the increase in hardness was 18 HV_0.2_. When the content of the reinforcement was increased to 30 wt%, microhardness also increased from 48 HV_0.2_ to 309 HV_0.2_, which was about 5.6 times that of the copper matrix. For the composite coating prepared under the condition of a higher energy density (70 kJ/cm^2^), when the reinforced phase content was 10 wt%, the hardness of the composite coating (89 HV_0.2_) was 1.6 times higher than that of the original copper substrate (55 HV_0.2_). The microhardness of the composite coating increased rapidly with a further increase in the reinforcing phase content. When the content was 30 wt%, the microhardness of the coating reached 256 HV_0.2_, which was about 4.7 times that of the copper substrate. The in-situ synthesized ceramic reinforcing phase (micron-scale primary ZrB_2_, nano-scale secondary ZrB_2,_ and nano-scale particle SiC) was dispersed in the coating, and through component strengthening: load transfer, dispersion strengthening, thermal mismatch strengthening, grain refinement, etc. [23,24,25]. This mechanism exhibited a higher composite strengthening effect, and thus the strength of the coating was increased significantly.

### 4.5. Tribological Properties of Composite Coatings

The composite coating was prepared under the optimized process parameters with an energy density of 60 kJ/cm2. The change in wear rate is represented by the following formula:

*W = kPV/H* (8)

where *W* is the wear rate of the material, *k* is the wear coefficient, *P* is the load, *V* is the sliding speed, and *H* is the hardness of the material. Therefore, the wear rate is proportional to the load *P* and sliding speed *V* and is inversely proportional to the hardness *H* of the material.

**Figure 12:**
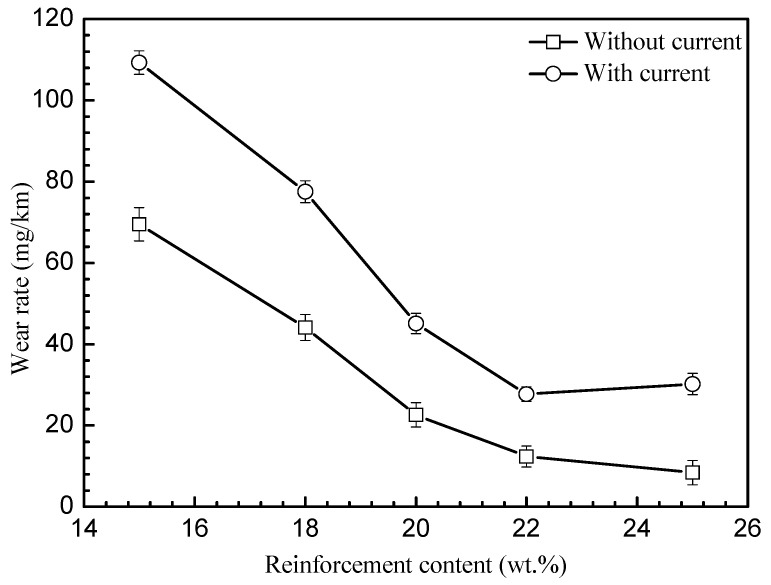
The wear rate of samples under different reinforcement contents and current loading.

Figure 12 shows the relationship between the wear rate of samples and the content of reinforced phases under different current conditions. It was observed that the wear rate changed under the condition of being with or without a current, following the same trend. When the content of reinforcements was increased, the wear rate decreased, and the decline rate of the wear rate decreased. A sharp decrease was seen when the content was increased from 15% to 22%; with a further increase in the contents to 25%, small changes in the wear rate were identified. The wear rate with a current was higher than that without a current. In addition, when the content of the reinforced phase was 15%, the wear rate was the highest, which was 68 mg/km and 109 mg/km under the conditions without/with a current, respectively. When the content of the reinforcing phase increased to 22%, the wear rate decreased to 30 mg/km and 14 mg/km and was maintained at similar values with a further increase in the content to 25%.

The reinforced phases in the composite coating were dispersed in the copper matrix and could play a significant role in the pinning enhancement during wear. Under the action of wear stress between the friction pairs, the addition of the harder phase distributes in the matrix increased the resistance of wear. The higher the reinforced phase content, the better the strengthening, so the cladding coating closer to the shear stress of materials obtained a lower wear rate. However, when a current was applied, the joule heat was generated because of the resistance between the friction pairs, which were superimposed with the heat generated by the tribological process and the heat impact was aggravated on the friction surface. Under the conditions of high-speed and the current load, the wear rate increased compared to that without current loading.

**Figure 13:**
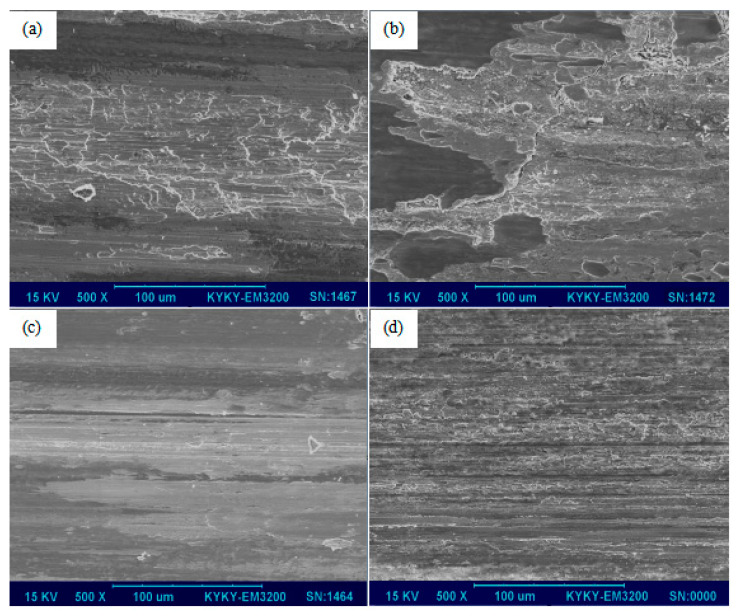
The wear surface morphology of cladding layer with different reinforcement phase contents and current-carrying. (**a**) 15%, 0 A; (**b**) 15%, 20 A; (**c**) 25%, 0 A; (**d**) 25%, 20 A.

The wear surface topography is shown in Figure 13, where Figure 13a,c shows the wear surface topography at the reinforcement phase content of 15, 25 wt% without a current. It could be observed that the surface of the coating with a 15% reinforcement was rugged and surface peeling in most of the surface area occurred at this time. The surface was flat for a composite with reinforcement at 25%, and shallow and long furrow scratches began to appear. The main reason for the above phenomenon was that when the content of the reinforced phase was lower (15%), the surface hardness of the coating was also lower, and a large amount of heat was generated in the friction process to soften the material surface. During the wear, the surface’s coating was seriously damaged by the opposite grinding plate, so the surface wear was serious and showed serious adhesive wear. When the content of the reinforced phase was higher (25%), surface hardness was also increased, strips of wear debris were deposited on the surface, and wear surface topography tended to level off into abrasive wear. The wear rate decreased with the increase of the reinforcement content, and the wear mechanism changed from adhesive wear to abrasive wear.

Figure 13b,d show the wear surface morphology of the coating composites at a 15% and 25% reinforcement content with a loading current of 20 A, respectively. The overall change trend was the same as the one without a current, but the wear became intensified under the same load and speed, accompanied by electric ablation, and cracks appeared when the content was 25%. The reason was perhaps not only that a large amount of joule heat was generated but also that the electrical arc ablation happened on the surface. The material’s surface was oxidized at the higher temperature. Some brittleness cracks were produced. The wear mechanism was also changed from a serious peeling wear to a slight peeling wear and abrasive wear together.

**Figure 14:**
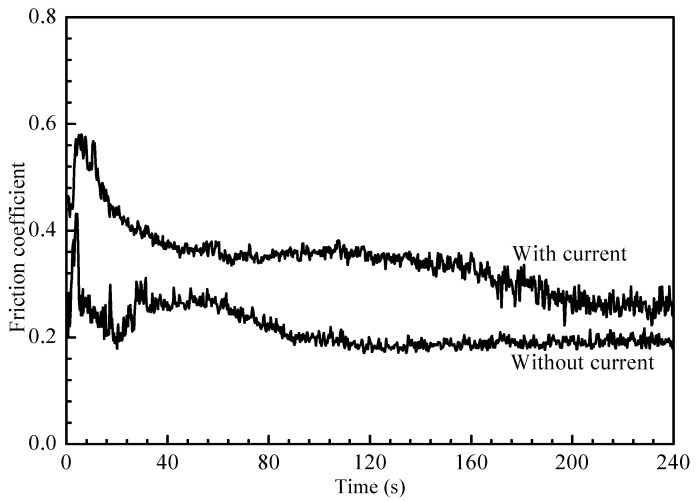
Friction coefficient curve under the condition of being with or without current.

The friction coefficient curves against time under the condition of being with or without a current are shown in Figure 14. It can be seen that a sharp rise in the friction coefficient followed a rapid decline in the initial stage. This was due to contact between the friction sample and the friction pair: initially, the actual contact area was relatively small, and the tip of the sample wear was large, so it appeared to be a high-friction coefficient, but the duration of this stage was relatively short (<20 s), as the wear continued. As the surface of the coating was flattened, the friction coefficient decreased gradually until the surface of the coating was flattened to a relatively stable wear stage, and the change in the friction coefficient became flat (average 0.4 or 0.2 with/without a current, respectively). It can also be seen from the diagram that it took less time to achieve stability under the current conditions. This was due to the softening effect of the current on the coating, so it took a shorter time to balance, and the friction coefficient was easier to stabilize. The friction coefficient fluctuated in the final stage of the case of current-carrying (160 s later) due to the action of the current, which resulted in the coating contact surface softening phenomenon. At the same time, strengthened phase particles were worn because of the continuous wear, and the surface of the coating became uneven owing to the falling off of the strengthened phase particles, so there was the phenomenon of friction coefficient fluctuation. In the case of non-current carrying, there was no arc erosion and melting action; therefore, the corresponding wear was relatively weak, long-term stability could exist, and the friction coefficient was basically maintained at a certain level (about 0.2). The wear rate was higher with the current than that without the current. The ZrB_2_-SiC composite coating had high wear resistance and electric ablation resistance.

## 5. Conclusions

(1). ZrB_2_-SiC/Cu composites underwent in-situ synthesis successfully onto the surface of the pure copper matrix by laser cladding.
(2). Under the condition of a laser energy density of 60 kJ/cm^2^ with the reinforced phase content of 30 wt%, the surface of the composite coating was continuously flat, and no defect was observed at the interface.
(3). The reinforcement morphology and microstructure in the coating were different. From the surface layer to the bottom, the size of the micron-scale ZrB_2_ gradually increased.
(4). The wear rate decreased with an increase in the reinforcement content, and the wear mechanism changed from adhesive wear to abrasive wear.
(5). The wear rate was higher with the current than that without the current. The ZrB_2_-SiC composite coating had a high wear resistance and electric ablation resistance.

## Figures and Tables

**Table 1:** Parameters of cladding powders.

Powder	Cu	Zr	Si	Ni Package B_4_C
Morphology	Spherical	Spherical	Irregular shape	Irregular shape
Particle size (μm)	≤75	≤48	≤75	≤25
Purity (%)	≥99.9	≥99.5	≥99.9	99.9

**Table 2:** Effect of energy density on the roughness, thickness, and interface of cladding coating.

E (kJ/cm^2^)	46.67	53.33	60.00	66.67	73.33
Roughness (Rv)	579	635	355	314	461
Thickness (mm)	2.1	1.9	1.8	1.5	1.3
Interface	good interface bonding	good interface bonding	good interface bonding	interface defects	dilution effect was serious

**Table 3:** Size of the reinforcement and microhardness of the composite under different laser energy densities.

Energy Density (kJ/cm^2^)	Size of SiC (nm)	Size of ZrB_2_ (μm)	Microhardness (HV_0.2_)
60	25	35	184
70	40	80	167

## Data Availability

Data sharing is not applicable to this article.
